# Jack of all trades: Genome assembly of Wild Jack and comparative genomics of *Artocarpus*

**DOI:** 10.3389/fpls.2022.1029540

**Published:** 2022-12-12

**Authors:** Ajinkya Bharatraj Patil, Sai Samhitha Vajja, S. Raghavendra, B. N. Satish, C. G. Kushalappa, Nagarjun Vijay

**Affiliations:** ^1^ Computational Evolutionary Genomics Lab, Department of Biological Sciences, Indian Institute of Science Education and Research (IISER), Bhopal, Madhya Pradesh, India; ^2^ College of Agriculture Hassan, University of Agricultural Sciences (UAS), Bangalore, Karnataka, India; ^3^ College of Forestry, Ponnampet, Karnataka, India

**Keywords:** Wild Jack, *Artocarpus*, Breadfruit, Jackfruit, Western Ghats, gene family evolution, positive selection, lineage-specific selection

## Abstract

*Artocarpus* (Moraceae), known as breadfruits for their diverse nutritious fruits, is prized for its high-quality timber, medicinal value, and economic importance. Breadfruits are native to Southeast Asia but have been introduced to other continents. The most commonly cultivated species are *Artocarpus heterophyllus* (Jackfruit) and *Artocarpus altilis* (Breadfruit). With numerous smaller but nutritionally comparable fruits on a larger tree, *Artocarpus hirsutus*, also called “Wild Jack” or “Ayani”, is an elusive forest species endemic to Indian Western Ghats. In this study, we sequenced and assembled the whole genome of *Artocarpus hirsutus* sampled from the sacred groves of Coorg, India. To decipher demographic and evolutionary history, we compared our Wild Jack genome with previously published Jackfruit and Breadfruit genomes. Demographic history reconstruction indicates a stronger effect of habitat rather than phylogeny on the population histories of these plants. Repetitive genomic regions, especially LTR Copia, strongly affected the demographic trajectory of *A. heterophyllus*. Upon further investigation, we found a recent lineage-specific accumulation of LTR Copia in *A. heterophyllus*, which had a major contribution to its larger genome size. Several genes from starch, sucrose metabolism, and plant hormone signal transduction pathways, in *Artocarpus* species had signatures of selection and gene family evolution. Our comparative genomic framework provides important insights by incorporating endemic species such as the Wild Jack.

## Introduction

Genus *Artocarpus* (Moraceae), or “Breadfruits,” are tropical plants famous for their nectary and fleshy fruits (Jarrett, 1977). This genus comprises ~70 species with considerable variability in size, height, flower/fruit morphology, developmental processes, and functional properties (Zerega et al., 2010; Gardner et al., 2021). Most of the members of the genus provide a rich resource of food, timber, and other valuable products, popularising them in their native regions (Jagtap and Bapat, 2010; Xavier et al., 2014; Ragone, 2018). As a consequence of such properties, some species have been introduced to various parts of the world. The two most widely distributed domesticated species, *Artocarpus heterophyllus* (Jackfruit) and *Artocarpus altilis* (Breadfruit), currently have oriental distribution in the tropical and subtropical regions (Zerega et al., 2010; Williams et al., 2017). However, *Artocarpus* trees are native to the region extending from the Western Ghats, South-East Asia, to the Oceanic Islands. Although a recent study suggested the diversification of *Artocarpus* from Borneo followed by subsequent dispersal and divergence during the Miocene (Williams et al., 2017), multiple fossils from India dated to the Palaeocene suggest an earlier presence (Mehrotra et al., 1984; Srivastava, 1998). Despite being unlikely, the Bornean origin of *Artocarpus* suggests overwater or overland dispersal across large distances as the only possibility for Indian *Artocarpus* species to exist (Williams et al., 2017). Hence, the biogeographical history of these plants is yet to be established and is a matter of further research. Differences in the bioclimatic properties of their habitats and the fauna involved in their pollination/dispersal might have played an instrumental role in adapting these species by developing divergent characteristics from their ancestral counterparts.

**Figure 1:**
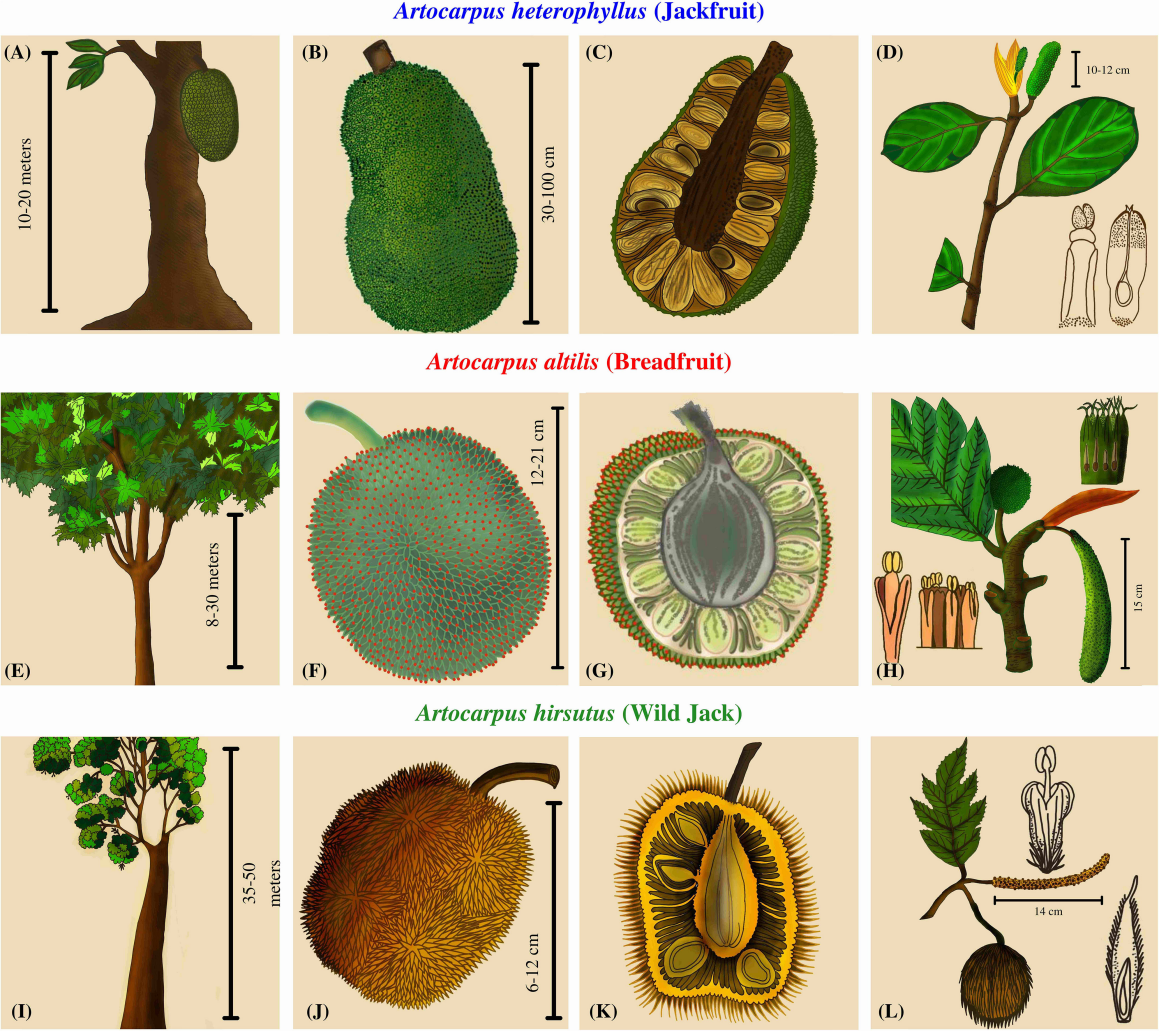
Plant morphology and comparative phenotypic characters of three *Artocarpus* species. **(A, E, I)** The first column depicts the overall structure of the trees. **(B, F, J)** The second column contains drawings of the fruit describing the color, shape, and size. **(C, G, K)** The third column has a cross-sectional view of the fruits depicting the number and arrangement of seeds. **(D, H, L)** The fourth column has drawings of the inflorescences.

*Artocarpus* trees are well known for their diversity of unique unisexual inflorescences and composite syncarpous fruits (Jarrett, 1977). The phenotypic diversity among the syncarps is such that the taxonomy of this genus is entirely dependent upon inflorescence morphology and structure (Zerega et al., 2010). Even though the focus has been on the floral diversity for delineating these species, these plants have evolved several other species-specific characteristics. The trees of *A. heterophyllus* reach a height of 15-20 meters and have reticulate branching close to the soil, whereas the trees of *A. altilis* reach up to 30 meters and are moderately branched at a medium height from the ground (**Figure 1**). As opposed to these two, Wild Jack (*Artocarpus hirsutus*) are large forest trees that reach above 50 meters, some extending to 70 m with no branching until the apices. The male inflorescences differ in all three species. *A. heterophyllus* has smaller cylindrical inflorescence than *A. altilis*, which has longer and thicker apices. In contrast, *A. hirsutus* has a thin, long, filamentous stalk and the male inflorescence differs entirely from the other two species. Female inflorescences also differ in these three species, so their fruit morphology is quite diverse. Jackfruit (*A. heterophyllus*) bears multiple, low-hanging, larger, ellipsoidal, fleshy, nectary, and green-sheathed fruits of sizes up to 100 cm. The Breadfruit (*A. altilis*) bears numerous medium-sized, oval, starchy, and green-sheathed fruits of 12-20 cm, hanging at apices of branches of medium heights. In comparison, Wild Jack (*A. hirsutus*) bears multiple oval/ellipsoidal, fleshy, smaller, orange/yellow sheathed fruits of size 6-10 cm at the apices of branches of higher heights. Therefore, such diverse phenotypic characteristics suggest differentiated pollinator/disperser networks and mechanisms (Jarrett, 1977; Matthew et al., 2006; Jagtap and Bapat, 2010; Ragone, 2018; Buddhisuharto et al., 2021).

The Wild Jack (*A. hirsutus*) is unique in its phenotype compared to the other two popular *Artocarpus* species. Due to its endemic distribution in the Western Ghats and its forests, it has received minimal attention and is still understudied (Matthew et al., 2006). However, in its native range, it is a multipurpose plant of economic and ecological importance. It provides long, high-quality, pathogen-resistant timber and has been widely used for building houses, boats, and large, long-lasting structures (Matthew et al., 2006; Xavier et al., 2014; Meenu et al., 2021). The other parts, like fruits and seeds, are used as a rich source of energy, and the constituents of this fruit are comparable or superior to the other two species, which demands further research into this plant (Solanki et al., 2020). Lastly, leaves, seeds, and bark are used in traditional medicinal treatments (Matthew et al., 2006; Jagtap and Bapat, 2010; Solanki et al., 2020; Buddhisuharto et al., 2021; Meenu et al., 2021). Wild Jack reaches maturity for timber harvesting in around 20 years, and the present distribution of the species cannot fulfill the increasing demand of the timber market, which makes it vulnerable to population decline. Hence the re-assessment of conservation status and efforts to effectively conserve this plant is warranted (Matthew et al., 2006; Liu et al., 2020). The sacred groves of the Western Ghats are fragmented forests protected by locals due to religious importance. But recently, these forests have been threatened due to deforestation and developmental projects (Matthew et al., 2006; Osuri et al., 2014; Wang et al., 2020). The biodiversity hotspot of the Western Ghats is home to multiple endemic flora and fauna (Myers et al., 2000), but there is a paucity of genomic data from these species. One such species is the Wild Jack, a ubiquitous constituent of these ancient forests conserving this species (Chandrashekara and Sankar, 1998; Tambat et al., 2005). By generating genomic resources, our study aims to incorporate Wild Jack in a comparative framework with other *Artocarpus* species. Hence, our goal is to identify genomic changes related to differential phenotypes and acclimatization to their habitats.

The phenotypic characteristics of *Artocarpus* species are quite distinct including their inflorescence structures, which tend to be associated with adaptive changes (Harder and Johnson, 2009; Harder and Prusinkiewicz, 2013). To uncover the genomic basis of phenotypic diversity between *Artocarpus* species, we require genomic information of Wild Jack to compare it with other genomes. Lineage-specific gene family changes and/or signatures of selection are potent drivers of phenotypic evolution and adaptation (Lespinet et al., 2002; Van Der Lee et al., 2017). Similarly, repeat accumulation can also lead to lineage-specific phenotypes (Negi et al., 2016; Li et al., 2018; Wang et al., 2018; Ramakrishnan et al., 2021). Therefore, we

a) Sequence and assemble the Wild Jack genome (and plastome), i.e., *Artocarpus hirsutus*.
b) Perform gene annotation to identify orthologous gene sequences across order Rosales members and construct a species tree.
c) Analyse gene family evolution in these species, especially in *Artocarpus* members.
d) Employ multiple methods to detect the signatures of selection in the genes of all *Artocarpus* species.
e) Detailed repeat sequence annotation to understand repeat accumulation dynamics.We wanted to evaluate the role of differential bioclimatic history on the demographic trends of species from differing habitats and whether it will be strongly affected by the ecological differences. We chose phylogenetically related species from distinct habitats to address this question. Hence, we
a) Use demographic reconstructions to analyse population size history and
b) Species distribution modelling to assess species range dynamics for *Artocarpus* species.

## Materials and methods

### Sample collection, genome sequencing, and assembly

*A. hirsutus* is endemic to the Western Ghats and its forests. We located a fruit-bearing tree near the College of Forestry, Ponnampet (GPS coordinates 12°08′56.5″N 75°54′32.5″E; Altitude: 829–850 m Above Sea level) and sampled some leaves for the sequencing. The samples collected for sequencing are preserved and cataloged (ART_HIR_WG_IISERB). A photograph of the leaf specimen is provided in **Supplementary Figure 1**. A leaf was cleaned, sanitised, and then cut into pieces for further processing. The whole genomic DNA was extracted from the leaves using DNeasy plant mini kit from QIAGEN. The quality of the extracted DNA was evaluated by observing the DNA band on 1% Agarose gel for shearing. The concentration and purity of extracted DNA were assessed using QUBIT 3.0 and Nanodrop. The purified DNA was then used to prepare Illumina short-read (150 bp) libraries with TruSeq DNA Nano Library Prep Kit with an insert size of 450 ± 50 bp. We sequenced ~ 88X coverage of the genome with Illumina short-read paired-end data using the Illumina Novaseq 6000 sequencer.

The quality of whole-genome sequencing (WGS) paired-end reads was assessed using FASTQC. Barcode sequences were trimmed if present using Cutadapt (Martin, 2011). These sequencing reads were used for the estimation of genome size. We used Jellyfish (Marçais and Kingsford, 2011) to perform Kmer analysis using a kmer-size (k) of 21 and hash size (-m) of 100M on the sequencing reads. GenomeScope (Vurture et al., 2017) was then used to estimate genome size and heterozygosity. We used the Celera assembler implemented in MaSuRCA version 4.0.6 (Maryland Super Read Cabog Assembler) for assembling the sequencing data (Zimin et al., 2013). The published assemblies of *A. heterophyllus* and *A. altilis* were used as a reference for the synteny-assisted assembly step of MaSuRCA. We used Quast (Gurevich et al., 2013) to calculate genome assembly metrics such as N50 and L50 (**Supplementary Table 1**). BUSCO version 3 (Simão et al., 2015) was used to assess the genome completeness with the eudicotyledons_odb10 dataset (**Supplementary Table 2**).

### Repeat annotation and analyses

For *de-novo* identification and annotation of the repetitive genomic regions and/or transposable elements, we used RepeatModeler version 2 (Flynn et al., 2020), with the LTR_struct option to include LTR models identified by programs such as LTR-FINDER (Xu and Wang, 2007) and LTR-Harvest (Gremme et al., 2013). The consensus fasta library obtained by RepeatModeler 2 was then used as input to RepeatMasker (Smit, AFA, Hubley, R & Green) to annotate, mask and tabulate the repeat content and their types. The resultant output file was then used to soft mask the genome for further analyses. The RepeatMasker.align output was used to calculate Kimura two-parameter divergence estimates (TE age) between the repeat families for all species using accessory scripts provided with RepeatMasker suite like buildSummary.pl, calcDivergenceFromAlign.pl, and createRepeatLandscape.pl. The obtained output was summarised to plot histograms of Kimura divergence values to visualise the distribution of repeat families across the time scale. The genome size of the species from order Rosales was correlated with the percent repeat content in their assemblies. To further nullify the effect of phylogenetic relatedness on the correlation, a correction was done using the PIC (Phylogenetic Independent Contrast) method implemented in the R package phytools.

### Genome annotation

We used MAKER version 2 (Campbell et al., 2014) to annotate the genome’s coding regions. Three rounds of the maker pipeline were executed to obtain the final annotated genesets. In the first round of homology-based annotation, we used protein fasta sequences from all the species of order Rosales available on NCBI, including *A. altilis*, and *A. heterophyllus* (**Supplementary Table 3**). The mRNA evidence from *A. altilis* was provided as alternative transcript sequences. The obtained genesets from this round were then used to generate training gene models for *de-novo* gene annotation algorithms like SNAP (Johnson et al., 2008) and AUGUSTUS (Stanke and Morgenstern, 2005). New gene models were identified during both rounds, and existing gene models were refined. Genesets after the third round were considered final and used to get coding sequences and translated protein sequences. We performed BUSCO on the resultant protein dataset to assess the quality of the annotations. We further used EggNOG functional annotation algorithm to get the gene names and GO annotations. We also used blastp with ARAPORT 11 database to validate the gene models.

### Chloroplast assembly, annotation, and analysis

The chloroplast sequence was independently assembled using WGS reads with NOVOPlasty version 4.3.1 (Dierckxsens et al., 2017). The chloroplast genome sequence of *A. altilis* (NCBI accession: NC_059002.1) was used as a reference for the algorithm, and the Maturase K gene sequence of *A. hirsutus* (NCBI accession: KU856362.1) was used as a seed, which is used as assembly generation point. The resultant assembly produced two contigs with only one arrangement possibility leading to a complete circular genome sequence spanning ~162Kbp. The chloroplast assembly was then annotated using GeSeq (Tillich et al., 2017), and the circular genome was depicted and visualised using OGDRAW (Greiner et al., 2019) implemented in CHLOROBOX. Currently available chloroplast genomes from the *Artocarpus* genus and outgroup species *Ficus religiosa* and *Morus indica* were downloaded from NCBI. To investigate rearrangements between these chloroplast genomes, they were aligned with ProgressiveMauve aligner (Darling et al., 2010) and visualised in Mauve alignment viewer (Darling et al., 2004). To identify the phylogenetic positions of these genomes, we aligned the genomes using the MAFFT aligner (Katoh et al., 2002). The appropriate substitution model was estimated using Modeltest-ng (Darriba et al., 2020), and the phylogenetic tree was constructed using Raxml-ng (Kozlov et al., 2019) with 1000 bootstraps. The chloroplast genomes of *A. heterophyllus* and *A. integer* show an inversion for the SSC (Small Single Copy) region compared to other *Artocarpus* sp. plastomes (**Supplementary Figure 2**).

### Identification of orthologous sequences and construction of species tree

The translated coding sequences of *A. hirsutus* and 13 other species (*A. altilis, A. heterophyllus, Morus notabilis, Parasponia andersonii, Trema orientale, Cannabis sativa, Rhamnella rubrinervis, Ziziphus jujuba, Malus baccata, Malus domestica, Prunus persica, Fragaria vesca,* and *Rosa chinensis*) were concatenated and used to find orthologs. We used Orthofinder (Emms and Kelly, 2019) to find orthologous genic sequences across 14 species with parameters to use MSA alignments to obtain the orthogroups using diamond blast (Buchfink et al., 2021), MAFFT (Katoh et al., 2002) and fasttree (Price et al., 2009). The orthologous gene sequences in which *A. hirsutus* is present were tabulated. These gene IDs were used to get corresponding CDS sequences from each species. These CDS sequences for all the genes were then aligned using GUIDANCE version 2 (Sela et al., 2015) with the MAFFT aligner (Katoh et al., 2002). All the resultant CDS alignments were concatenated and used to find partitions and models using IQTREE version 2 (Minh et al., 2020). After that, the loci and concatenated trees were obtained to get bootstrap support with additional metrics such as the Gene concordance factor (gCF) and Site concordance factor (sCF). Following these evaluations, the tree was exported and rooted at a branch leading to *F. vesca* and *R. chinensis*.

### Comparative genomics and gene family analyses

The translated coding sequences of 4 species, *A. hirsutus, A. altilis, A. heterophyllus*, and *M. notabilis* were used to identify overlapping and non-overlapping gene clusters using Orthovenn version 2 (Xu et al., 2019). Orthovenn identified and constructed the unique and common gene clusters for all four species. Unique gene clusters of *Artocarpus* species were subjected to GO enrichment analysis. We used CAFÉ version 5 software (Mendes et al., 2021) for gene family analyses of contractions and expansions. We first concatenated protein sequences of 14 species used for species tree construction and made a blast database. This 14-species protein database was used as a subject to perform all vs. all protein blast (blastp) (Camacho et al., 2009). The blast results were then used as input for mcxload to create network and sequence dictionary files. The clustering was performed using mcl clustering software (Li et al., 2003) with an inflation parameter (-I) of 3. The cluster files were then reformatted, and the gene families with large gene copy numbers were removed from the analyses. The constructed species tree was converted to an ultrametric tree using r8s software (Sanderson, 2003) using a divergence estimate of 87 MYA (Million Years Ago) between *P. persica* and *Z. jujube* obtained from TimeTree (Kumar et al., 2017). The filtered clustering file of MCL and the ultrametric tree were then used as input for the CAFÉ 5. The clade-based gene family expansion/contraction results were then summarised and represented on the phylogeny. Out of all significant gene family contractions, we selected only those gene families with a difference of five gene copies at the least between the species. By enforcing these stringent criteria, we got seventeen, three, and seven gene families significantly expanded in *A. hirsutus*, *A. heterophyllus*, and *A. altilis*, respectively.

### Lineage-specific selection tests in *Artocarpus* genes

Tests of selection intensity among species for the same orthologous genes in a phylogenetic framework provide opportunities to identify loci under relaxed or intensified pressures in a focal species of interest. This selection pressure analysis helps us identify evolutionary changes and signs of adaptations to their bioclimatic niche. We used multiple approaches to identify selection pressures in *Artocarpus* to understand the evolutionary mechanisms and processes these species have undergone. We used branch-site models implemented in PAML version 4.9 (Yang, 2007) and aBSREL (Adaptive Branch-site Random Effects Likelihood) (Smith et al., 2015) implemented in HYPHY to test for positively selected branches. We also used RELAX (Wertheim et al., 2015) (intensification parameter; K > 1) implemented in HYPHY to identify the genes under intensified selection. For detecting strong purifying or relaxed selection, we implemented the branch site model of PAML version 4.9 and RELAX (relaxed parameter; K < 1) of HYPHY. To reduce the false positive results, we compared the list of genes identified as positively selected by all three methods and considered only those genes that were overlapping/common between them. The functional roles of positively selected genes were cross-referenced using KEGG (Kanehisa and Goto, 2000), FLOR-ID (Bouché et al., 2016), ARAPORT11 (Cheng et al., 2017), and TAIR (Rhee et al., 2003) databases.

### Demographic history reconstruction

The genomic sequencing reads of one individual each of *A. hirsutus*, *A. altilis*, and *A. heterophyllus* were mapped to the respective genome assemblies using the BWA MEM aligner (Li, 2013). The alignments were converted to binary, sorted, and indexed using samtools (Li et al., 2009). These binary alignments were then used to call consensus sequence using bcftools (Li and Barrett, 2011). To assess the effect of genomic regions such as exonic, intronic, intergenic, and repetitive elements on the demographic estimation, we masked each part to evaluate the impact of the respective fraction. We masked the respective genomic region using BEDTOOLS maskfasta and followed similar steps mentioned above to get the demographic estimation. To assess the effect of each individual repeat family/type, we followed the published protocol/scripts (Patil and Vijay, 2021). We quantified the concordance between the trajectories estimated from different repeat types within each species using a non-parametric measure of intraclass correlation implemented in the “nopaco: Non-Parametric Concordance Coefficient” R package (Rothery, 1979). We calculated the difference in N_e_ estimates between the Unmasked and Masked trajectories and the differences between the Unmasked and each repeat type. Using the difference between unmasked and masked trajectories as the maximum deviation in trajectories, we evaluated which repeat types had a similarly large deviation from the unmasked estimates. For this, we performed Wilcoxon tests between the (Unmasked-masked) and (Unmasked-each repeat type) (see **Supplementary Table 4**). The repeat types with significant differences are not major contributors to the masked estimates. Therefore, the comparisons with non-significant p-values between (Unmasked-masked) and (Unmasked-each repeat type) are the repeats that have contributed the most to the deviation from unmasked estimates.

We used filters like -C50, -Q30, -q20 for bcftools mpileup to ensure quality bases and mapped reads to be considered in the variant calling. The consensus calls were converted to the required (fastq) format using vcfutils vcf2fq using a quality filter of 25, whereas calls with less than one-third and more than twice the mean coverage were excluded during this step to exclude false calls. These consensus calls were then converted to input format (.psmcfa) for psmc using fq2psmcfa. The input psmcfa file was then used to run the psmc program (Li and Durbin, 2011) with options -N 25 -t 5 -r 5 -p 4 + 25*2+4+6. The output of psmc was inspected for a sufficient number of recombination events. At first, we used a mutation rate (µ) of *Populus trichocarpa*, i.e., 2.5e-09 per site per year (Tuskan et al., 2006) with a generation time of 15 years to execute the psmc_plot.pl script to get scaled demographic trajectories for each species.

### Estimation of mutation rate

Since we were trying to study demographic effects on these three species comparatively, we needed to understand the bottleneck events for these species from their native ranges. *A. hirsutus* and *A. heterophyllus* are native to the Western Ghats and should have experienced similar demographic events. Our initial scaled PSMC plots for both species with the same mutation rates did not align with the starting point of the trajectory. These trajectories created a possibility that there might be mutation rate differences between these three species. To obtain a reliable mutation rate estimate, we sampled orthologous alignments in which only four species (*A. hirsutus, A. altilis, A. heterophyllus*, and *M. notabilis*) are present. We fixed an input un-rooted tree structure to allow branch-specific comparisons possible. We used codon alignments of ~1500 genes to estimate a (d_4_) 4-fold degenerate site substitution rate (parameters used, model = 0, NSsites=0, seqtype=1, CodonFreq=2, runmode=0) using PAML version 4.9. The obtained d_4_ rates for all alignments were summarised, and the mean value for these estimates was considered d_4_ for individual species. These mean d_4_ estimates were then divided by the divergence time between compared branches or species. The estimates obtained were then considered a proxy of the respective species’ mutation rates (Nadachowska-Brzyska et al., 2015).

### Species distribution modelling

We downloaded species occurrence data corresponding to the native range of each species as identified earlier (Williams et al., 2017) from the GBIF (Global Biodiversity Information Facility) database for all three *Artocarpus* species (Gbif.Org, 2022). We used the method of Ecological niche modelling (ENM) to predict the species distribution during three paleoclimatic eras: Last Glacial Maximum (LGM, approx. 20,000 years ago), Last interglacial (LIG, approx. 110,000-130,000 years ago), and Marine Isotope Stage 19 (MIS19, approx. 750,000-790,000). The environmental variables for these periods were extracted from PaleoClim (Brown et al., 2018) at a resolution of 2.5 min arc. Environmental layers were resized to the species’ native range using the software DIVA-GIS (version 7.5) (Hijmans et al., 2012). We considered the annual and excluded the seasonal parameters for highly correlated bioclimatic variables. The set of variables used was chosen based on species-specific considerations for the compared periods (see **Supplementary Tables 5B-C**).

The ENM was performed using the software MaxEnt (version 3.4.4). The settings for MaxEnt were species and paleoclimatic era-specific. We used the R package ENMeval, which identifies settings that balances model fit and increases the predictive ability (see **Supplementary Table 5E**) (Muscarella et al., 2014). The following settings were set by default: 10000 background points, 500 maximum iterations, ten runs of cross-validations, and the regularisation multiplier were explicitly based on ENMeval results. We saved the output in cloglog form, which is the simplest to understand and the default output format. It gives the probability of occurrence estimate between 0 to 1. We selected the mean of all ten replicate runs to represent each species across each time period. The average of the population count across these 10 runs was calculated as the Population Count (PC). The number of grid cells with a habitat suitability index > 0.9 was calculated as the Grid cell Count (GC). The accuracy in the prediction of species distribution was analysed through the use of a receiver operating characteristics (ROC) plot. In the ROC plots, all the values fell between 0 and 1 (AUC: Area Under the Curve). All the values were above 0.5 and are considered better than random when the curve lies above the diagonal, indicated by the AUC (see **Supplementary Table 5A**) (Merow et al., 2013). A Jackknife test was performed to find the different contributions of variables and to identify the ones with a maximum contribution (see **Supplementary Table 5D**). The habitat suitability maps of species distribution were generated using R.

## Results

### Genome sequencing, assembly, and annotation

The whole genome sequencing of Wild Jack (*A. hirsutus*) yielded ~475 million Illumina short reads (71.65 Gigabases). The genome assemblies of previously published congeneric species vary from ~800 Mbp (*A. altilis*) to ~980 Mbp (*A. heterophyllus*); their Kmer-based genome size estimates are 812 Mbp and 1005 Mbp, respectively (Sahu et al., 2019). The 21 Kmer value-based genome size estimate for *A. hirsutus* is 635.16 Mbp with 1.16% heterozygosity, a smaller genome size estimate than other two congenerics. The resultant genome assembly was 791.16 Mbp in length. Our resultant assembly captures nearly complete genomic information for *A. hirsutus* as it is substantially greater than the Kmer-based estimate but closer to the nearest congeneric, *A. altilis*. The generated genome assembly has coverage of ~ 90X. The assembly has a contig N50 of 50.25 Kbp with L50 of 4630. Our assembled genome has 96.7% of complete BUSCO’s, indicative of a nearly complete assembly. More than 98% of the sequencing reads were mapped to the genome assembly. The LTR-Retriever’s LAI score for the assembly was 6.28, and indicates it is a draft genome assembly. The MAKER pipeline annotated 46,957 gene models with a mean length of 2387.62 bp. BUSCO identified 94.6% of protein gene sets from the MAKER as complete, which indicates good annotation and an almost complete gene set.

### Lineage-specific gene family dynamics

**Figure 2:**
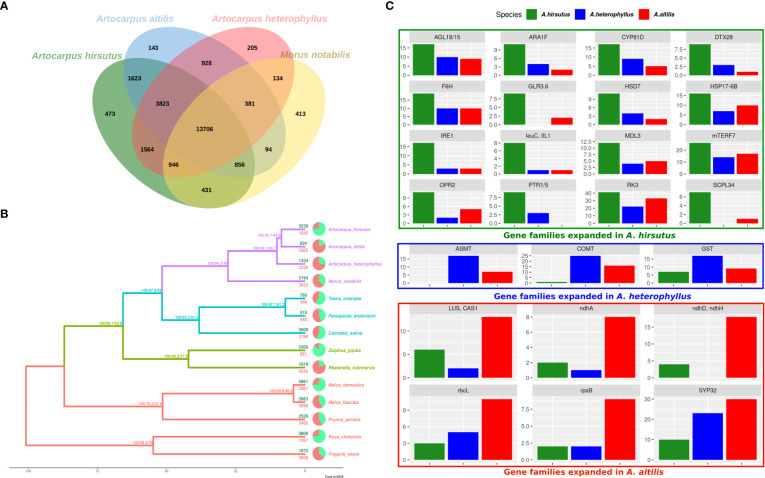
Comparative genomics of orthologous gene content evolution in *Artocarpus* and their relatives. **(A)** Orthovenn diagram of three Artocarpus species (*A. hirsutus, A. altilis,* and *A. heterophyllus*) with *M. notabilis*. Unique and shared gene clusters between these species are denoted in respective intersections. *A. hirsutus* has 473 unique gene clusters not shared with other congenerics. **(B)** Representative species tree with gene family evolution or Gene gain-losses: 14 species from Order Rosales of family Moraceae (purple), Rhamnaceae (sea green), Cannabaceae (olive green), and Rosaceae (red) are represented in the species tree based on ~4500 orthologous genes. Branch values are bootstraps values, gCF (gene concordance factors) and sCF (site concordance factors), respectively. Pie charts and associated numbers at the tips show gene families’ expansions (green) and contractions (red). **(C)** Significantly expanded gene families in *A. hirsutus* (green), *A. heterophyllus* (blue), and *A. altilis* (red).

Gene family expansion and diversification are prominent drivers of phenotypic evolution. Comparative analysis of *Artocarpus* genomes and outgroups from Rosales’ order identified changes in gene family composition (see **Figures 2A, B** and **Supplementary Tables 6, 7**). Notably, lineage-specific genes identified by orthovenn2 in *A. hirsutus* are enriched for pollen recognition genes (GO: 0048544), which are Receptor Kinases from the Lectin domain-containing gene family (**Supplementary Table 7**). We also found evidence of lectin gene family expansion based on CAFÉ analysis in *A. hirsutus* (41 copies) compared to *A. altilis* (33 copies) and *A. heterophyllus* (22 copies) (see **Figure 1C**, RK3 panel, **Supplementary Table 8**). Among the lectin genes with orthologs across all three *Artocarpus* species, we detect signatures of intensified selection using RELAX and positive selection using the PAML branch site and aBSREL (**Supplementary Figure 3**). Lectin domain-containing proteins have diverse functions in biotic and abiotic stress response, plant growth, and development (Sun et al., 2020; Saidou and Zhang, 2022). Therefore, our results suggest diversification of lectin domain-containing proteins in *A. hirsutus*.

Apart from lectins, *A. hirsutus* also showed lineage-specific gene family expansions in at least 15 other gene families with functions varying from pollen/flower development (AGL18/15, ARA1F, PTR3, EDA17), secondary metabolite biosynthesis (F6’H, IIL1, HSD7, DTX28 (Upadhyay et al., 2020), MDL3), stress tolerance and defence, i.e., biotic (IRE1, IIL1, DTX28, MDL3) and abiotic (F6’H, CYP81D8, GLR3.6, SCPL34, PTR1/5, HSP17-6B, OPR2), growth and development (mTERF7, HSD, AGL18/15, ARA1F) and plant-pathogen interactions (IRE1, F6’H, IIL1, OPR2, MDL3) (see **Figures 2C**). All these numerous gene family expansions may reflect the concerted evolution of this plant to acclimatise to biotic and abiotic conditions and adapt to its habitat.

In *A. heterophyllus*, the lineage-specific genes identified by orthovenn2 are enriched for Toll-Interleukin-Resistance (TIR) domain proteins, Receptor Like Protein 33 (RLP33), and the flavonoid biosynthesis pathway. TIR domain proteins and RLP33 are well known for foreign pattern recognition and providing immunity to plants from microbes (Burch-Smith and Dinesh-Kumar, 2007; Jamieson et al., 2018). In addition, the two most essential genes of the Flavanoid Biosynthesis Pathway, Chalcone Synthase (CHS) and Flavanone 3-Hydroxylase (F3H) have lineage-specific gene copies and may explain the high flavonoid content of *A. heterophyllus* (Meera et al., 2018). The copy number of both ASMT (N-Acetylserotonin Methyltransferase) and COMT (Caffeic Acid O-methyltransferase) genes is higher in *A. heterophyllus* (17 and 25 copies) compared to *A. hirsutus* (0 and 1 copies) and *A. altilis* (7 and 16 copies) (**Supplementary Figure 4**). ASMT and COMT genes act in the penultimate step of the melatonin pathway (Back et al., 2016; Zhao et al., 2019). Furthermore, COMT also plays an important role in the lignin biosynthesis pathway (Wang et al., 2013). Lastly, the gene family of Glutathione S-Transferases (GST), which have a role in stress tolerance, has also expanded in *A. heterophyllus*.

The lineage-specific genes of *A. altilis* are enriched for Hexokinase-3, ABCB27 (ATP-Binding Cassette B27) or ALS1 (Aluminium Sensitive 1), and mTERF15. Hexokinase-3 is involved in sugar processing, primarily glucose and plant development (Paulina Aguilera-Alvarado and Sanchez-Nieto, 2017). ABCB27 or ALS1 are transporters involved in stress response to Aluminium-rich or Acidic soils (Kar et al., 2021). The transcription factor mTERF15 modulates the expression of mitochondrial assembly factor I genes, specifically NAD2/3 (NADH ubiquinone oxidoreductases), and regulates energy generation (Hsu et al., 2014). Interestingly, we found gene family expansions in multiple genes of mitochondria and chloroplast (ndhH, ndhD, ndhA, rbcL, and rpsB) in *A. altilis*. These expansions of organellar genes and their regulators might be due to a higher energy requirement caused by oxidative stress or other stressors. The triterpenoid biosynthesis synthase genes like Cycloartenol Synthase (CAS1) and Lupeol synthase 2/5 (LUP2/5) (Thimmappa et al., 2014; Cárdenas et al., 2019) and essential pollen development proteins, syntaxin of plants (SYP31/32) (Rui et al., 2021) were also increased in copy number.

### Habitat rather than phylogeny determines the population histories

**Figure 3:**
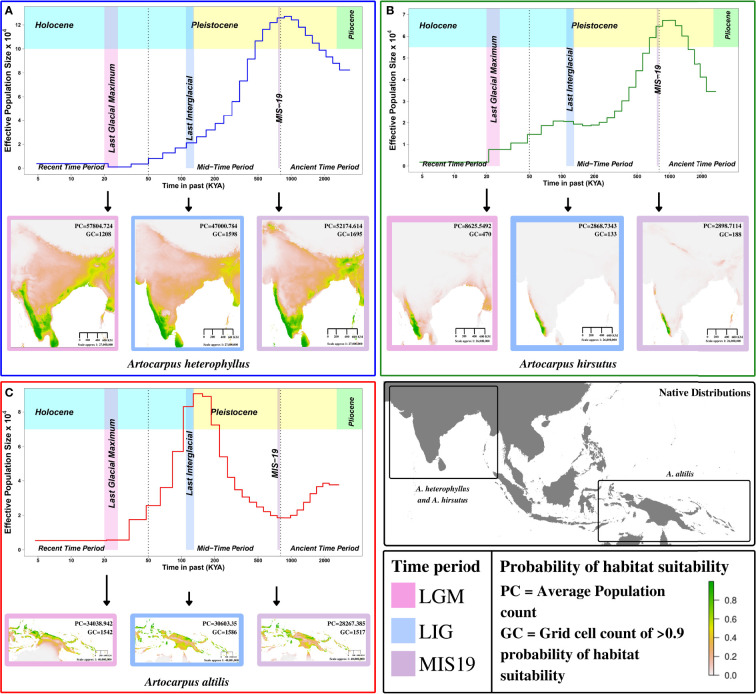
Demographic history reconstruction and species distribution modelling of *Artocarpus* species. Demographic history reconstruction using PSMC with Species distribution modelling for LGM (Last Glacial Maximum), LIG (Last Interglacial), and MIS19 (Marine Isotope Stage 19) for **(A)** *A. heterophyllus* (blue), **(B)** *A. hirsutus* (green), **(C)** *A. altilis* (red). The two dotted vertical lines are used to demarcate the recent, mid and ancient time periods following the timeline shown. The native distribution of all three species are highlighted in the bottom right map of the continent.

**Figure 4:**
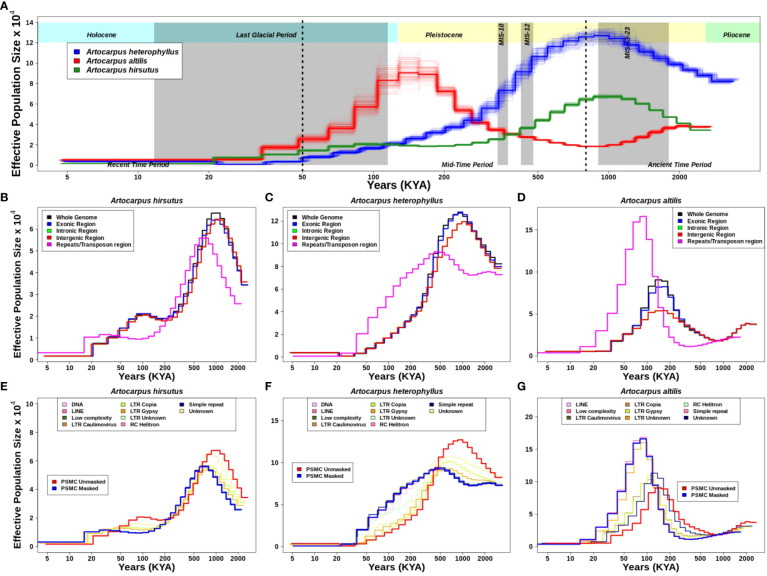
Demographic history reconstruction of three *Artocarpus* species. **(A)**. Demographic history of *A. heterophyllus* (blue), *A. altilis* (red), and *A. hirsutus* (green) are represented by the respective trajectories with additional bootstrapped lines. The two dotted vertical lines are used to demarcate the recent, mid and ancient time periods following the timeline shown. The grey vertical shading represents the corresponding glacial event. Demographic trajectories and the effect of genome fraction used for PSMC inference in **(B)**. *A. hirsutus*, **(C)**. *A. heterophyllus*, and **(D)**. *A. altilis*. The effect of different repeat types on PSMC inference is represented in **(E)**. *A. hirsutus*, **(F)**. *A. heterophyllus*, and **(G)**. *A. altilis*. The amount of change due to LTR-Copia is most prominent in *A. heterophyllus*.

We found that *A. altilis* underwent demographic contraction ~ 2 to 1 million years ago (MYA), followed by extensive population expansion from ~ 1 MYA to 150 thousand years ago (KYA), which marks the start of the Holocene or Last Glacial Period (**Figures 3C**, **4A**). The demographic expansion of *A. altilis* from MIS-19 to the LIG is accompanied by an increase in habitat suitability (i.e., GC increases from 1517 to 1586 and PC increases from 28267.39 to 30603.35). In contrast to *A. altilis*, the population sizes of *A. heterophyllus* and *A. hirsutus* experienced extensive expansion from ~ 2 to 1 MYA, followed by a contraction in population size from ~ 1 MYA to 200 KYA (**Figures 3A, B**, **4A**). The demographic contraction in both species from MIS-19 to the LIG is accompanied by corresponding reductions in habitat suitability for *A. heterophyllus* (i.e., GC decreases from 1695 to 1598 and PC decreases from 52174.614 to 47000.784) and *A. hirsutus* (i.e., GC decreases from 188 to 133 and PC decreases from 2898.7 to 2868.7343). After the onset of the Holocene, the effective population size declined in *A. altilis* and *A. heterophyllus*. However, in *A. hirsutus* the population size recovered and stabilised before undergoing another round of population decline in the Holocene. The discrepancy between SDM and PSMC in the recent time period might be due to inability of PSMC to reliable estimates in this time period. Comparing demographic histories in the mid and ancient time periods among the three *Artocarpus* species and the species distribution models suggests that bioclimatic changes and the habitat have been instrumental in shaping the population histories. In conclusion, the demographic histories of the *Artocarpus* species reflect the effects of habitat more than their phylogenetic relatedness (**Figure 4A**).

The estimates of historical effective population size (N_e_) reflect evolutionary processes such as actual changes in population size, population structure, gene flow (Mazet et al., 2015, 2016), and linked selection (Schrider et al., 2016) and/or regions of the genome used (Patil and Vijay, 2021). Hence, we evaluated the effect of different genomic regions in estimating demographic histories. Exon, intron, and intergenic region-masked trajectories matched with the whole-genome-based trajectory, which explains that these individual regions of the genome are not drastically changing the estimates (**Figures 4B-D**). However, masking repetitive genomic regions resulted in two types of changes in the inferred trajectory. The less noticeable trajectory change results in a diagonal shift towards recent time intervals in *A. hirsutus* and *A. altilis*. The more drastic change in trajectory occurs in *A. heterophyllus*, where the repeat masked and whole-genome-based N_e_ estimates have a lower concordance. The measures of concordance between the trajectories estimated from different repeat types were higher for *A. altilis* (0.915) and *A. hirsutus* (0.942) compared to *A. heterophyllus* (0.872). The pairwise differences in the concordance between species found that *A. altilis* and *A. hirsutus* did not differ significantly (p-value: 0.07). However, the comparison of both (*A. altilis* vs. *A. heterophyllus*: delta is 0.0465 and p-value is 0.000553 and *A. hirsutus* vs. *A. heterophyllus*: delta is 0.0723 and p-value is 1.86e-05) these species with *A. heterophyllus* has significant differences. Overall, our results indicate that repeats have influenced the demographic inferences of *A. heterophyllus* (concordance between masked and unmasked genomes psi: 0.783 and p-value: 1.83e-05) more than the other two species (*A. altilis*; psi:0.94 and p-value:1.18e-30, *A. hirsutus*; psi:0.892 and p-value:3.4e-18).

To understand which type of repeat regions affect the inference of demographic history in these species, we investigated the effect of each repeat type. In all three species, the shift in trajectories among LTRs (i.e., LTR-Unknown, LTR-Gypsy, and LTR-Copia) was highest (**Figures 4E-G**). Other repeat families, like simple repeats, DNA transposons, low complexity regions, etc., mirrored the masked trajectory and had no effect of masking on demography. Unknown repeat types had the most noticeable impact on the trajectories of *A. hirsutus* (Wilcoxson test p-value: 0.03513) and *A. altilis* (Wilcoxson test p-value: 0.07581), whereas LTR-Copia (Wilcoxson test p-value: 0.9934) greatly impacted the N_e_ estimates of *A. heterophyllus*. The most surprising result of masking repetitive regions occurs in the N_e_ estimates of *A. heterophyllus*, which drastically changes the trajectory in magnitude and shape mainly due to LTR-Copia.

### Differential abundance/accumulation of repeat families in *A. heterophyllus*

**Figure 5:**
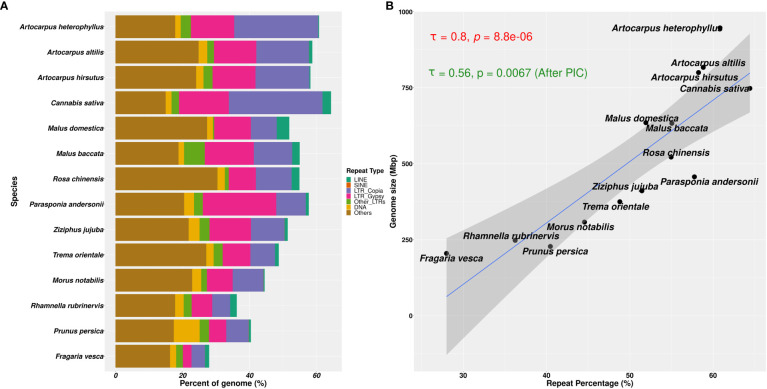
Repetitive genomic regions and their abundance in Rosales genomes. **(A)** Repeat content percentages in genomes of 14 species of Rosales with the abundance of types of repeats across these species. **(B)** Correlation of genome sizes with percentage of repeat content assembled in these 14 genomes. There is a strong positive correlation (τ = 0.8 and p-value = 8.8e-06, after Phylogenetically Independent Contrasts (PIC): τ = 0.56 and p-value = 0.0067) between the percentage of repeat sequences assembled in the genome and the assembly sizes.

We found that the repetitive genomic regions strongly affected the demographic analyses, which demands further detailed characterisation of repeat families and their contents. We compared the types of repeat families assembled in those 14 Rosales genomes and their abundances (see **Figure 5A**). Of the three *Artocarpus* genomes, *A. hirsutus* (481 Mbp; 60.51% of the genome) and *A. altilis* (505 Mbp; 60.68% of the genome) have a comparable composition of repeat types. In contrast, *A. heterophyllus* (614 Mbp; 62.56% of the genome) has a higher overall repeat content than the other two species. Specifically, the abundance of LTR-Copia in the *A. heterophyllus* genome (246.5 Mbp; 25.1% of the genome) was highly elevated compared to *A. altilis* (131 Mbp; 15.7% of the genome) and *A. hirsutus* (128 Mbp; 16.1% of the genome). Other than LTR-Copia, most other families, except for some unknown/unannotated LTRs, were similarly abundant across the three *Artocarpus* species. These differences in repeat composition suggest a species-specific expansion or excessive accumulation of LTR-Copia family repeats in *A. heterophyllus*. Among the *Artocarpus* outgroup genomes, *C. sativa* has LTR-Copia and overall repeat content expansion similar to *A. heterophyllus*.

Genome size evolution is a product of various factors, including the repetitive profile of the species. Repeat sequence accumulation can inflate the genome size of a species and shape genome evolution. To address if repeat expansions and assemblages in the genomes of Order Rosales significantly impacted their genome sizes, we correlated their total assembly sizes (i.e., a proxy for genome size) and percent of repeat content. We observed a strong positive correlation between the percent repeat content in these genomes with their genome sizes (**Figure 5B**, Kendall’s correlation coefficient = 0.8, p-value = 8.8e-06; after PIC correction, Kendall’s correlation coefficient = 0.56, p-value =0.0067). The strong correlation suggests that Order Rosales underwent genome size evolution strongly influenced by repeat expansions and accumulations.

**Figure 6:**
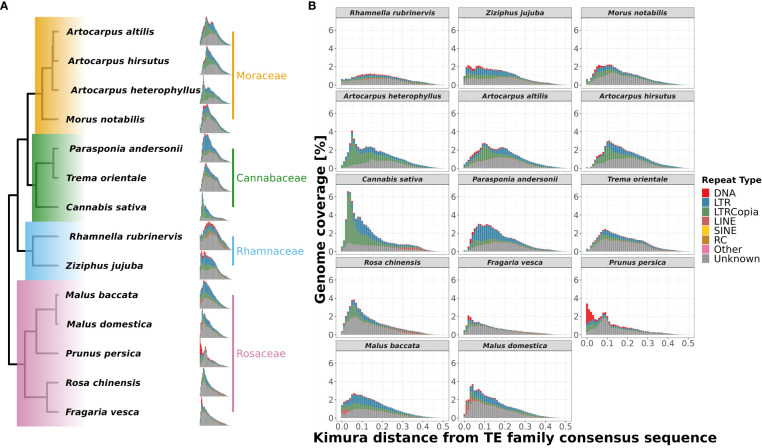
Repeat landscape evolution in 14 species of order Rosales. Family-wise changes in repeat landscapes: **(A)** The differences in abundance and insertion ages of repeat families are depicted phylogenetically. The recent spike in the fraction of the genome covered by repeats of *A. heterophyllus* suggests a recent accumulation not shared by the other two species of *Artocarpus*. **(B)** Repeat landscapes for 14 species of order Rosales showing the distribution of different repeat types and their ages of insertions depicted by Kimura distance (along the x-axis) and Genome coverage (along y-axis) percentages. *A. heterophyllus* shows a peak (3.5% of genome coverage) of LTR-Copia abundance during the recent period (~0.05 Kimura distance), which is not shared by the other two species as they have a peak (~2.5% of genome coverage) during the comparatively older period (~0.1 Kimura distance).

To understand the differential species-specific repeat accumulation in the order Rosales, we used Kimura two-parameter divergence estimates to reconstruct the timeline of repeat expansion (see **Figure 6**). All three *Artocarpus* species have a comparable repeat abundance of ~2% genomic content at a Kimura distance of ~0.1, and this likely represents their shared history of repeat accumulation. However, *A. heterophyllus* has recently accumulated species-specific repeats corresponding to ~3.5% genomic content, primarily LTR-Copia sequences at a Kimura distance of ~0.05. Hence, the recent accumulation of the LTR-Copia is most likely the reason for genome size expansion in *A. heterophyllus* after divergence from *A. altilis* and *A. hirsutus*. Like *A. heterophyllus*, *C. sativa* also has a similar pattern of recent LTR-Copia repeat accumulation compared to other members of Cannabaceae. Rosaceae family has a rich diversity of plants with flowers and fruits with economic and commercial value. Plants of this family prove to be diverse in terms of species-specific repeat-type accumulation. For instance, *Malus* species have a recent expansion of LINE sequences.

Similarly, *F. vesca* and *P. persica* have an unusual abundance of DNA CMC repeats but have accumulated at different Kimura distances. While the repeat content in *F. vesca* has peaked at a Kimura distance of ~0.05, the accumulation of repeats in *P. persica* appears to be more recent. Further research is required to understand if this represents an ongoing insurgence of DNA CMC by comparing high-quality genomes and transcriptomes of closely related species/varieties.

### Species-specific signatures of selection

To reduce false positives, we used genes identified as positively selected by the three approaches (i.e., PAML, aBSREL, and RELAX). While this approach identifies a smaller set of genes, these results are more reliable and robust to the approaches employed (see **Supplementary Figure 5**). We discuss the pathways with several positively selected genes in a comparative framework to understand putative species-specific adaptations (for the complete list of genes, see **Supplementary Tables 9-11**).

#### Starch and sucrose pathway

**Figure 7:**
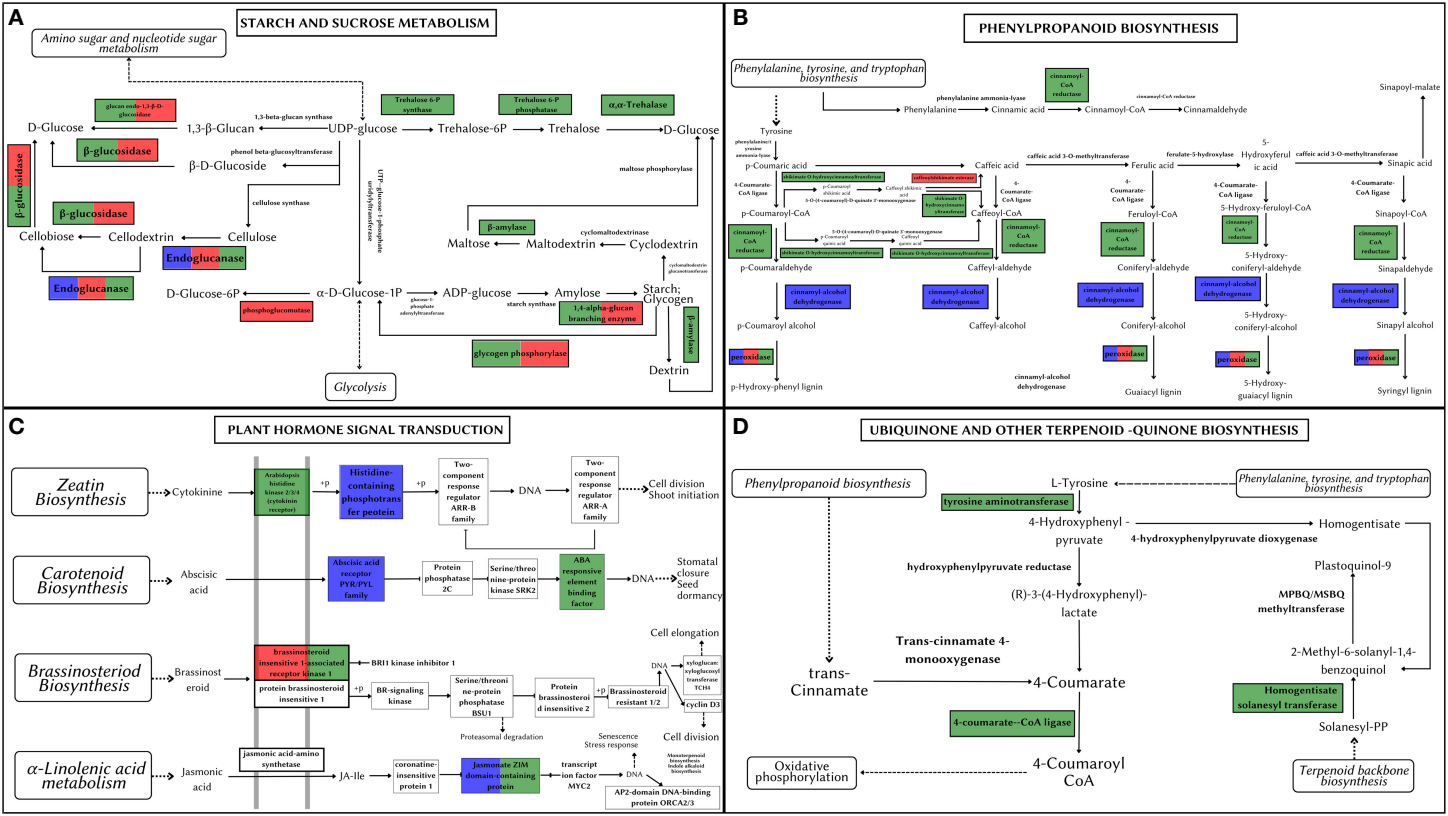
Metabolic pathways depicting positively selected genes in *Artocarpus* species. The positively selected genes in *A. hirsutus* (green), *A. heterophyllus* (blue), and *A. altilis* (red). **(A)** Starch and Sucrose Metabolism. **(B)** Phenylpropanoid biosynthesis. **(C)** Plant hormone signal transduction. **(D)** Ubiquinone and other-terpenoid-quinone biosynthesis.

Starch and sucrose metabolism is at the heart of plant growth and development. All three *Artocarpus* species shared signatures of positive selection in genes producing (1) GBE1 (1,4 alpha-glucan branching enzyme) involved in the Starch synthesis step and (2) Cellulase/endoglucanase involved in the breakdown of cellulose (see **Figure 7A**). The genes coding for BGLU (Beta-Glucosidase) and EGLC (Glucan endo-1,3-beta-D-glucosidase) were positively selected in both *A. hirsutus* and *A. altilis*. These genes are involved in synthesizing D-glucose by producing multiple intermediate metabolites. However, there are multiple species-specific shifts in selection strength among the three *Artocarpus* species. For instance, *A. hirsutus* has multiple positively selected genes in different subprocesses of the starch and sucrose metabolism pathway and includes all the genes involved in the conversion of UDP-glucose to D-glucose through the production of Trehalose-6-P and alpha-Trehalose, i.e., otsA (Trehalose 6-phosphate synthase), otsB (Trehalose 6-phosphate phosphatase) and TREH (Trehalase). The BAM (Beta-amylase) gene involved in the breakdown of starch into Dextrin and Maltose through Maltodextrin was also positively selected in *A. hirsutus*. Interestingly, none of these genes had any signatures of selection in the other two *Artocarpus* species. Similarly, *A. altilis* has a species-specific positive selection in the PGM (Phosphoglucomutase) gene involved in converting D-glucose-1-phosphate to D-glucose-6-P. In conclusion, the comparative analysis of positively selected genes in this pathway highlights the differential regulation of plant developmental processes, especially in *A. hirsutus*, which has several positively selected genes in Trehalose synthesis and metabolism.

#### Phenylpropanoid biosynthesis/lignin pathway

The phenylpropanoid pathway is involved in the biosynthesis of secondary metabolites such as lignins and flavonoids using Phenylalanine, tyrosine, and tryptophan-derived compounds. All three species of *Artocarpus* have signatures of positive selection in the genes producing peroxidase (PRX/PRDX) enzyme, which catalyses the last step of lignin biosynthesis by converting lignin alcohols to lignins (see **Figure 7B**). Species-specific positive selection is detected in *A. hirustus* among the genes involved in the pathway’s initial stages, such as 4CL (4-coumarate-coA ligase) and HCT (shikimate O-hydroxycinnamoyltransferase). Similarly, positive selection is detected in CAD (Cinnamyl-alcohol dehydrogenase) and CSE (Caffeoyl shikimate esterase) for *A. heterophyllus* and *A. altilis*, respectively. 4CL is common to both lignin and flavonoid biosynthesis pathways, while CAD, HCT, and CSE are considered lignin pathway-specific genes (Falcone Ferreyra et al., 2012; Yao et al., 2021). However, HCT is also thought to have a role in flavonoid biosynthesis (Ren et al., 2020).

#### Plant hormone signal transduction

Hormone signal transduction involves numerous crucial players in plant development. A transmembrane protein, BAK1 (Brassinosteroid insensitive 1-associated receptor kinase 1), is a co-receptor of BRI1 (Brassinosteroid insensitive 1) and plays a vital role in development, stress tolerance, and plant-pathogen interactions. BAK1 is positively selected in *A. hirsutus* and *A. altilis* but not in *A. heterophyllus*, suggesting differential developmental regulation in these species (see **Figure 7C**). Apart from BAK1, *A. hirsutus* has elevated selection pressure on another transmembrane protein HK2/3 (Histidine Kinase 2/3), the receptor for cytokinin, which is instrumental in shoot initiation and vascular bundle formation. ABF (ABA-responsive element binding factor) protein involved in regulating plant abiotic stress responses is also positively selected in *A. hirsutus*. Another important factor involved in JA (Jasmonic Acid) pathway, JAZ (Jasmonate ZIM domain-containing protein) (Pauwels and Goossens, 2011), also experiences higher selective pressure in *A. hirsutus* and *A. heterophyllus*. JA pathway is involved in almost every developmental process, including flower and root development and protection or response to multiple biotic or abiotic stress (Yang et al., 2019). Furthermore, *A. heterophyllus* also has two more genes that have elevated selection pressure, AHP (Histidine-containing phosphotransfer protein) and PYL (abscisic acid receptor PYR/PYL family) regulators of cytokinin and ABA (Abscisic acid), respectively.

#### Ubiquinone and terpenoid-quinone biosynthesis

Ubiquinone and other quinone-related compounds participate in multiple growth and developmental processes and act as antioxidants to provide stress tolerance (Liu and Lu, 2016). Genes involved in this pathway such as 4CL, TAT (Tyrosine aminotransferase), HST (Homogentisate solanesyltransferase), COQ6 (Ubiquinone biosynthesis monooxygenase), and NDC1 (Demethylphylloquinone reductase) all were positively selected in *A. hirsutus*, whereas neither of the two other *Artocarpus* species had any positive selection in this pathway (see **Figure 7D**).

#### Carotenoid biosynthesis

**Figure 8:**
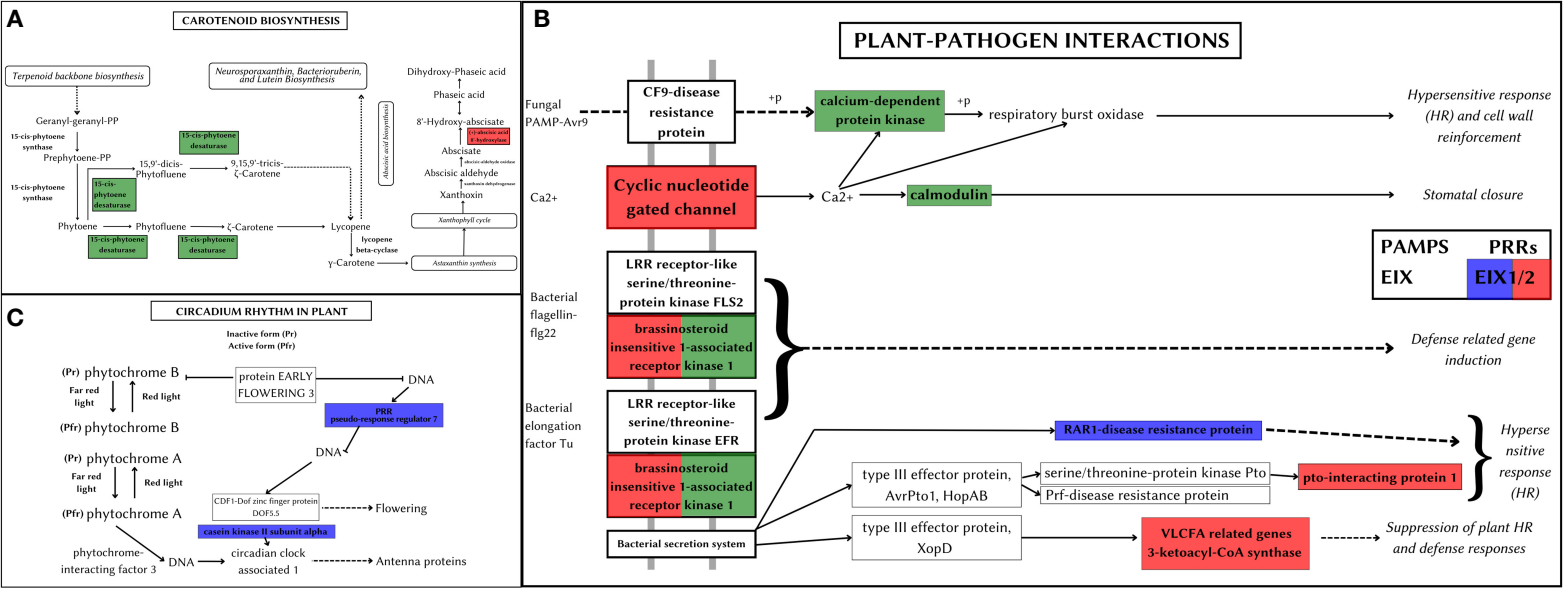
Metabolic pathways depicting positively selected genes in *Artocarpus* species. The positively selected genes in *A. hirsutus* (green), *A. heterophyllus* (blue), and *A. altilis* (red). **(A)** Carotenoid biosynthesis. **(B)** Plant-pathogen interactions. **(C)** Circadian rhythm in plants.

Carotenoid pathway aids in the development, stress response, and synthesis of carotenoid products (Shumskaya and Wurtzel, 2013). *A. hirsutus* has a positively selected gene PDS (15-cis-phytotene desaturase) involved in the production of carotenoids, specifically zeta-carotenoids and their derivatives. These carotenoids are yellowish and are involved in fruit ripening (Naing et al., 2019). The CYP707A gene, which is involved in catabolising ABA and its regulation, is positively selected in *A. altilis* (see **Figure 8A**). ABA is involved in germination and other stress responses, which suggests CYP707A may be regulating seed development processes (Kim et al., 2020).

#### Plant-pathogen interaction

Plant-pathogen interactions impact the survival and development of the plant. The plant can elicit pathogen-specific immune responses by assessing the nature of the pathogen. *A. hirsutus* has three positively selected genes involved in this pathway, CPK (Calcium-dependent protein kinase), CALM (Calmodulin), and BAK1 (see **Figure 8B**). CPK and CALM are part of fungal PAMP-triggered immunity (Pathogen-Associated Molecular Pattern) and provide fungi-specific responses. BAK1 is involved in the bacterial pathogen response of the plant. RAR1 protein (Disease resistant protein) involved in effector-triggered immunity against bacterial pathogens is positively selected in *A. heterophyllus*. The EIX receptor 1/2, which is a Pattern recognition receptor (PRR), was positively selected in both *A. heterophyllus* and *A. altilis*. Other than this PRR, *A. altilis* showed positive selection in multiple different genes involved in plant-pathogen interactions, like CNGC (Cyclic nucleotide-gated channel), a transmembrane protein providing fungal response PTI1 (pto-interacting protein 1), BAK1 and KCS (3-ketoacyl-coA synthase) involved in bacterial defence responses.

#### Circadian rhythm in the plant

Circadian rhythm of plants controls the molecular and cellular expression patterns to regulate better and mediate the light and dark periods, which in turn gives a fitness benefit to the plant (Venkat and Muneer, 2022). *A. heterophyllus* has two positively selected genes, PRR7 (pseudo-response regulator 7) and CSNK2A (Casein kinase II subunit alpha), which regulate plant circadian rhythms (see **Figure 8C**). The importance of efficient control of light and dark periods could also be an evolutionary adaptation that could have led *A. heterophyllus* to distribute to a larger geographical area.

#### Floral gene evolution

Floral genes AS1 (Asymmetric Leaves 1) and HUA2 (Enhancer of AG-4 2) experienced strong selection pressure in both *A. hirsutus* and *A. heterophyllus*. At the same time, TIL1 (Tilted 1), TPS1 (Trehalose-6-phosphate synthase), GA2 (GA requiring 2), and MEE27 (Maternal effect embryo arrest 27) were positively selected only in *A. hirsutus*. VIP5 (Vernalisation Independence 5) and PRR7 (Psuedo-response regulator 7) experienced elevated signatures of selection only in *A. heterophyllus*. PFT1 (Phytochrome and flowering time 1), HDA9 (Histone deacetylase 9), and LNK2 (Night light-inducible and clock-regulated 2) have lineage-specific signatures of selection in *A. altilis*. Similarly, PHYE (Phytochrome E) was positively selected in both *A. altilis* and *A. heterophyllus*.

All the genes that had functions in light signaling and/or circadian rhythm regulation were under strong signatures of selection in *A. altilis*, and few of them were in *A. heterophyllus*. This might be the case because of the dependence on the tight regulation of flowering time for these plants’ fitness. However, genes under strong selection pressure in *A. hirsutus* were involved in meristematic growth, fungal defense, pollen/flower development, and identity. Such processes explain the dependence on maintaining the phenotype rather than adapting to changes in light periodicity. But, *A. heterophyllus* experiences selection pressure on both circadian and other phenotype-related genes and hence might have allowed the evolution of a distinctive phenotype with a wider distribution.

## Discussion

Our newly generated Wild Jack genome allowed comparative genomics of phenotypically diverse, phylogenetically distant Breadfruit species from contrasting habitats. Changes in gene content between these species reflect putative modifications in the distinct phenotypes of these plants. Notably, several genes from the same biological processes, such as flower development and response to biotic/abiotic stress, have experienced changes in copy number and may have allowed the rewiring of these pathways. Signatures of selection also occur in some genes of these same pathways leading to fine-tuning the altered phenotypes. Whole-genome sequence data allows the reconstruction of the demographic history and comparison between closely related species. Hence, such reconstructions provide helpful insight into habitat-specific responses. We complemented the information from these genomics-based reconstructions with species distribution modeling. Broadscale patterns identified by species distribution models were concordant with the trajectory inferred by genomics-based reconstructions and indicated that Jackfruit and Wild Jack species, which share the same habitat, have a comparable population size history. In contrast, the Breadfruit, which has a native range with a history of volcanic eruptions, has a unique history of population bottlenecks. While the effect of habitat on the demographic history is expected, the response of each *Artocarpus* species to the habitat is idiosyncratic.

### How has the habitat shaped the genomes of *Artocarpus*

The demographic reconstruction and species distribution modelling revealed the effects of differentiated bioclimatic forces acting on their bottleneck history and distribution patterns in two diversified habitats, i.e., the Western Ghats (Jackfruit and Wild Jack) and East of Sulewasi (Breadfruit). The oceanic region of East Sulewasi islands has a history of volcanic eruptions, with accumulated ash resulting in acidic soils. These acidic soils have unique properties such as low phosphate, high iron, high Aluminium content, and other minerals or microelements. The climate and precipitation cycles are also different compared to the Western Ghats. In contrast, the Western Ghats have nutrient-rich, alkaline soils with abundant biotic meta-compositions of various taxa in the soil (Myers et al., 2000). This nutrient-rich soil contains numerous bacterial and fungal pathogens, and plants must adapt to achieve fitness in interacting or responding to these species. All these differences have impacted flora and fauna of these regions, and hence these plants must adapt to differential plant-biotic interactions. Breadfruit (*A. altilis*) has multiple gene family expansions in the OXPHOS assembly complex and chloroplast genes. We also identified lineage-specific copies of the organellar expression regulator transcription factor mTERF15 and acidic soil response transporter ABCB27. Therefore, due to harsh soil and bioclimatic properties, the mitochondrial assembly genes and their regulators in Breadfruit have experienced gene family expansion. These multiple expansions in energy-producing pathways can be explained by the higher energy demand of the plant to sustain oxidative stress response and acquire resistance to Aluminium-rich acidic soils.

Jackfruit and Wild Jack have multiple gene family changes related to plant-biotic interactions and secondary metabolite productions, which are important determinants for biotic and abiotic adaptations. The Wild Jack shows gene family expansions for IRE1, GRIP, SCPL-II, PTR1, DTX28, HSP20, MDL3, and receptor kinases, all involved in either biotic, abiotic stress tolerance/response, or plant immunity. Similarly, the Jackfruit has gene family expansion in the stress-related GST gene family and unique gene clusters of genes like the TIR domain gene involved in plant immunity and the RLP gene family involved in stress responses. In addition, both Western Ghat species have signatures of selection in anti-fungal genes such as AS1, DMS11 (Defective in meristem silencing 11), RD20 (Responsive to desiccation 20), LECRK-IX.1 (L-TYPE LECTIN RECEPTOR KINASE IX.1) conferring fungal-resistant properties to its timber.

### Why such divergent phenotypes among *Artocarpus* trees

The faunal consumers prefer the fruits of Jackfruit and Wild Jack as they are sweet, fleshy, and nectary. However, the Breadfruit is a starchy fruit that is not as sweet and nectary as the other two; hence it is eaten as a vegetable rather than fruit. As discussed above, due to higher energy expenditure to sustain oxidative stress response, many other pathways with relatively more minor functions might have been impacted, reduced, or relaxed and could explain the loss of the ancestral sweet and nectary fruit phenotype in Breadfruit. Although the distribution of these plants overlaps, the two Western Ghats species, Jackfruit and Wild Jack differ in their plant height, branching, fruit size, colour, etc, and their responses to similar biotic environments may have been different due to their contrasting growth patterns. Wild Jack is a typical forest-adapted species with large trees having unidirectional growth, maintaining the apical branching to compete for sunlight efficiently, and a strong tap root system to utilize water and nutrients in dense forests. Due to this phenotype of Wild Jack, the lineage-specific gene family expansions, unique gene clusters, and genes showing selection signatures are primarily attributed to plant-pathogen interactions, stress responses, and floral evolution.

The fruits of Wild Jack are at a greater height, reducing its niche of vertebrate land dispersers such as elephants, boars, and other ruminants. The increased height ensures a different mechanism for both pollination and dispersal. The long and stalky male inflorescences of Wild Jack, in contrast to the short cylindrical inflorescences of Jackfruit, might be a switch from faunal dependence for pollination to a wind-pollinated mechanism and can explain multiple gene family expansions, unique gene clusters, and positive selection in pollen recognition genes from the lectin gene family, the Receptor kinases. Due to the switch to wind pollination, the plant must have devised some mechanisms to maintain Self Incompatibility (SI). The receptor kinases are well known to function in maintaining SI to avoid self-pollination and allow cross-pollination as much as possible (Sherman-Broyles et al., 2007). The number of fruits is more and has distinctively bright yellowish-orange colour and smaller sizes as compared to others which is an adaptation for attracting birds, bats, and primates as their dispersers (Primack, 2003; Flörchinger et al., 2010). The Lion-tailed Macaque (*Macaca silenus*), endemic to the Western Ghats, is one of the most important consumers of these fruits and can be considered their dispersers (Kumara and Santhosh, 2013). Some hornbills have also been observed eating these fruits. These pollination/disperser-specific changes in Wild Jack might be due to gene family changes and stronger positive selection in floral genes like AGL15/18, ARA1F, PTR3, EDA17, RK3, TIL1, TPS1, GA2, MEE27, AS1, HUA2, etc. In comparison, the colour of the fruit could be due to strong selection pressure on carotenoid biosynthesis genes. For example, the positively selected gene PDS is crucial for synthesizing zeta-carotene, which has a yellowish pigment. All these genomic changes have translated into the phenotype of the Wild Jack to adapt to the forest habitat and fine-tune its pollination and disperser network.

Trehalose metabolism contributes to processes involving embryogenesis and various other processes (Lunn et al., 2014). Additionally, TPS1 regulates axillary bud outgrowth and modulation of axial shoot branching (Fichtner et al., 2021). In the Wild Jack genome, all the trehalose metabolism genes are positively selected, suggesting its importance in maintaining the phenotype of apical branching and changes in inflorescence structure. Moreover, Wild Jack has a gene family expansion in F6’H (Feruloyl-CoA 6’-hydroxylase) which catalyses the penultimate step in scopoletin synthesis, a simple coumarin. A recent study (Hoengenaert et al., 2022) demonstrated that elevated expression of Scopoletin in lignifying cells leads to higher production of monosaccharides. Due to the higher lignocellulosic mass of Wild Jack, the Trehalose pathway’s involvement in generating sugars and their conduction seems likely in this plant.

In contrast to Wild Jack, Jackfruit has a short, branched tree structure with low-hanging fruits that are not suited for dense forests. The large fruits of Jackfruit are nectary and sweet with inflorescences that also impart volatile compounds, which attract a species of Gall Midge that may facilitate pollination (Gardner et al., 2018). The low-hanging Jackfruit is consumed by large mammals like elephants, wild boar, and other ruminants, facilitating its dispersal. Specifically, effective long-range dispersal is possible due to the long-distance migration of these dispersers. Therefore, the unique phenotypes of Jackfruit allow efficient fauna-based pollination/dispersal mechanisms. Gene family expansions and lineage-specific selection among genes of the flavonoid biosynthesis pathway, like Chalcone-synthase, could have facilitated the evolution of nectary fruits and inflorescences with volatile compounds. The widespread distribution of Jackfruit spans regions with differing light periodicity. Hence, the need to adapt to these changes. The strong signatures of selection in the genes involved in light signaling or circadian rhythm suggest a tight regulation of light periodicity-related pathways. Consequently, an efficient plant-pollinator/disperser network and tight regulation of circadian rhythm might have played an instrumental role in maintaining Jackfruit’s wider distribution range and larger population size. Similar pollinator/disperser-influenced evolution of inflorescence has been established in closely related *Ficus* species (Zhang et al., 2020; Wang et al., 2021).

### Did LTR-Copia accumulation shape Jackfruit evolution

We observed recurrent genome size changes due to repeat content dynamics in Rosales’ order. We also see that the genomes of order Rosales show a strong positive correlation between their genome sizes and repeat content (**Figure 5B**). The increase in genome size with repeat content suggests that genome size evolution is influenced by repeat expansion. The size of the assembled *A. heterophyllus* (Jack fruit) genome (~980 Mb) is ~200Mb larger than that of *A. altilis* (~800 Mb) and *A. hirsutus* (~790 Mb). Assembled genome sizes concord with the K-mer-based estimates and is largely unaffected by assembly quality. Moreover, the genomes of *A. heterophyllus* and *A. altilis* are from a single study (Sahu et al., 2019) that uses the same methodology for sequencing and assembling both genomes, ensuring comparable genome quality. The difference in genome size among the *Artocarpus* species is primarily due to the increased prevalence (~150 Mb) of LTR-Copia in *A. heterophyllus (see* **Figure 5A**). The genome of *C. sativa* from the sister family Cannabaceae also has a larger genome, potentially due to a lineage-specific accumulation of LTR-Copia. Investigation of repeat accumulation dynamics suggests recent lineage-specific repeat expansions in these two phylogenetically distant species in a similar time frame, which suggests a role of habitat or stress-mediated induction of repeats. Repeat content change in plants has been linked to functional diversification through cis-regulatory changes or other epigenetic mechanisms (Negi et al., 2016; Hirsch and Springer, 2017). The conflict between transposable elements and the host defense mechanisms is elevated in stress conditions resulting in improved regulatory machinery (Wang et al., 2018). Hence, the accumulation of LTR-Copia in *A. heterophyllus* has played a pertinent role in the evolution of the Jackfruit genome. In future studies, gene expression data will allow the identification of ongoing transposon activity and its effect on gene regulation.

## Limitations and broader implications

Gene content tends to be underestimated in fragmented genomes, and genome quality heterogeneity can confound comparative genomics. All three *Artocarpus* genomes compared in this study have similar BUSCO scores and are fairly comparable in gene content. Additionally, we put forth multiple stringent criteria to avoid false positives. We identify several candidate pathways that have experienced changes in gene content and positive selection. Detailed functional characterisation of these candidates by evaluating changes in gene expression and the consequent phenotypic changes will require further studies. The occurrence data for *Artocarpus* is limited and influenced by human-mediated dispersal, which could confound the species distribution modelling. The single genome-based demographic history reconstructions performed using the PSMC method are known to be unreliable in the recent past (<20KYA). Future studies can provide better resolution by incorporating population-level sampling.

Of the ~70 species of *Artocarpus*, our study includes only three whole genomes. Although our study highlights the potential of such comparative genomic studies, the inclusion of multiple other species would be able to provide definitive answers to questions regarding the origin, phenotypic diversity, and diversification. For instance, the genetic basis of syncarp evolution in this genus can be explored to exploit the molecular mechanisms involved in achieving desired phenotypes. Such species-rich genera with heterogeneous phenotypes are especially well suited for agroforestry genomics (Feng and Du, 2022). Hence, *Artocarpus* can serve as a model to understand inflorescence/syncarp biology.

## Conclusion

Our study has generated genomic resources for a forest tree, the Wild Jack, which is endemic to the Western Ghats. This dataset will help understand the evolution of forests and fill a gap in sampling forest tree genomes. Comparative genomic analysis with other *Artocarpus* species and members of the order Rosales has provided interesting insights into their genomic evolution. For example, habitat-driven evolution through phenotypic diversification has resulted in genomic signatures of selection and gene-family changes. Similarly, the demographic history reconstructions from genomic data and species distribution modelling strongly support the prominent role of habitat. And lastly, the adaptive changes in plant growth and development, floral morphology, and biotic interactions have shaped the Wild Jack to thrive in the forests and may explain its endemism and current fragmented distribution. In contrast, Jackfruit and Breadfruit appear tightly regulated by light signalling and circadian rhythm leading to more widespread distribution. Additionally, the fruit morphology/sizes might be due to genic evolution in floral development and may be due to the habitat-specific rewiring of the pollinator/dispersal network. Our comparative genomic analysis of *Artocarpus* con-generics exemplifies genomic changes associated with phenotypic diversity and habitat-mediated demographic changes.

## Data availability statement

The data presented in the study are deposited in the ENA repository (https://www.ebi.ac.uk/ena), and the accession numbers are PRJEB55580 and ERZ12974505. Scripts and data are available at: https://github.com/Ajinkya-IISERB/Wild_Jack and https://doi.org/10.17632/vc6vwbrzs4.1.

## Author contributions

AP and NV wrote the manuscript with inputs from SV and CK, BS, and SR collected the samples required for primary data generation. AP analyzed the genomic data and generated all the results. Species distribution modeling analysis was done by SV, who also prepared the illustrations used in this manuscript. All authors contributed to the article and approved the submitted version.

## Supplementary material

Supplementary Figure 1 Leaf specimen of *A. hirsutus* (Wild Jack) sampled from the sacred groves, Kodagu, Coorg, Karnataka, India.

Supplementary Figure 2 Chloroplast genome of *A. hirsutus* and rearrangements in Genus Artocarpus. **(A)** The assembled chloroplast genome of *Artocarpus hirsutus* with a length of ~161 Kbp. **(B)** Whole plastome-based bootstrapped phylogeny of *Artocarpus* and outgroup species *Ficus religiosa* and *Morus indica*. The second panel shows Mauve alignment of variable regions of these chloroplast genomes. All *Artocarpus* genomes show a similar arrangement of inverted repeat and small single copy (SSC) regions except *A. heterophyllus* and *A. integer*. These two genomes show an inverted arrangement of sequences and genes of the SSC region.

Supplementary Figure 3 The phylogenetic relationship between gene copies of the Lectin gene family Receptor Kinase 3 (RK3). Gene copies of *Artocarpus hirsutus* are coloured in green, *A. heterophyllus* in blue, and *A. altilis* in red.

Supplementary Figure 4 Phylogenetic relationship between gene copies of ASMT and COMT gene families. The sky blue coloured cluster of genes is the COMT gene family, whereas the red coloured is ASMT. Gene copies of *Artocarpus hirsutus* are coloured in green, *A. heterophyllus* in blue, and *A. altilis* in red.

Supplementary Figure 5 Approaches used to detect positive and relaxed selection in *Artocarpus* species. Genes identified as positively selected by all the approaches are shortlisted as positively selected genes.
