# Supplementary material for: Jack of all trades: Genome assembly of Wild Jack and comparative genomics of Artocarpus
Source: Front Plant Sci. 2022 Dec 12;13:1029540. doi: 10.3389/fpls.2022.1029540 (PMC9791056; doi:10.3389/fpls.2022.1029540)

Supplementary Figure S1

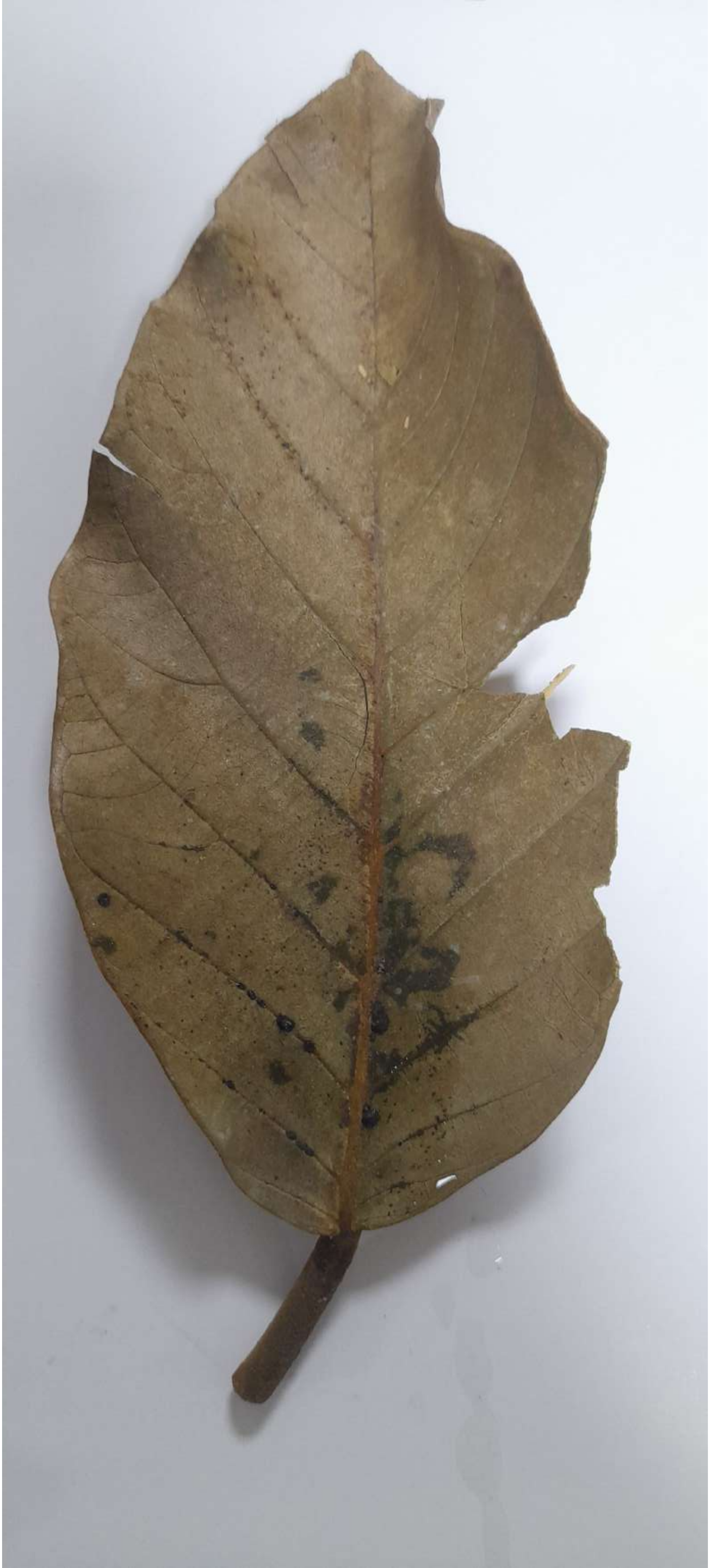

Supplementary Figure S2

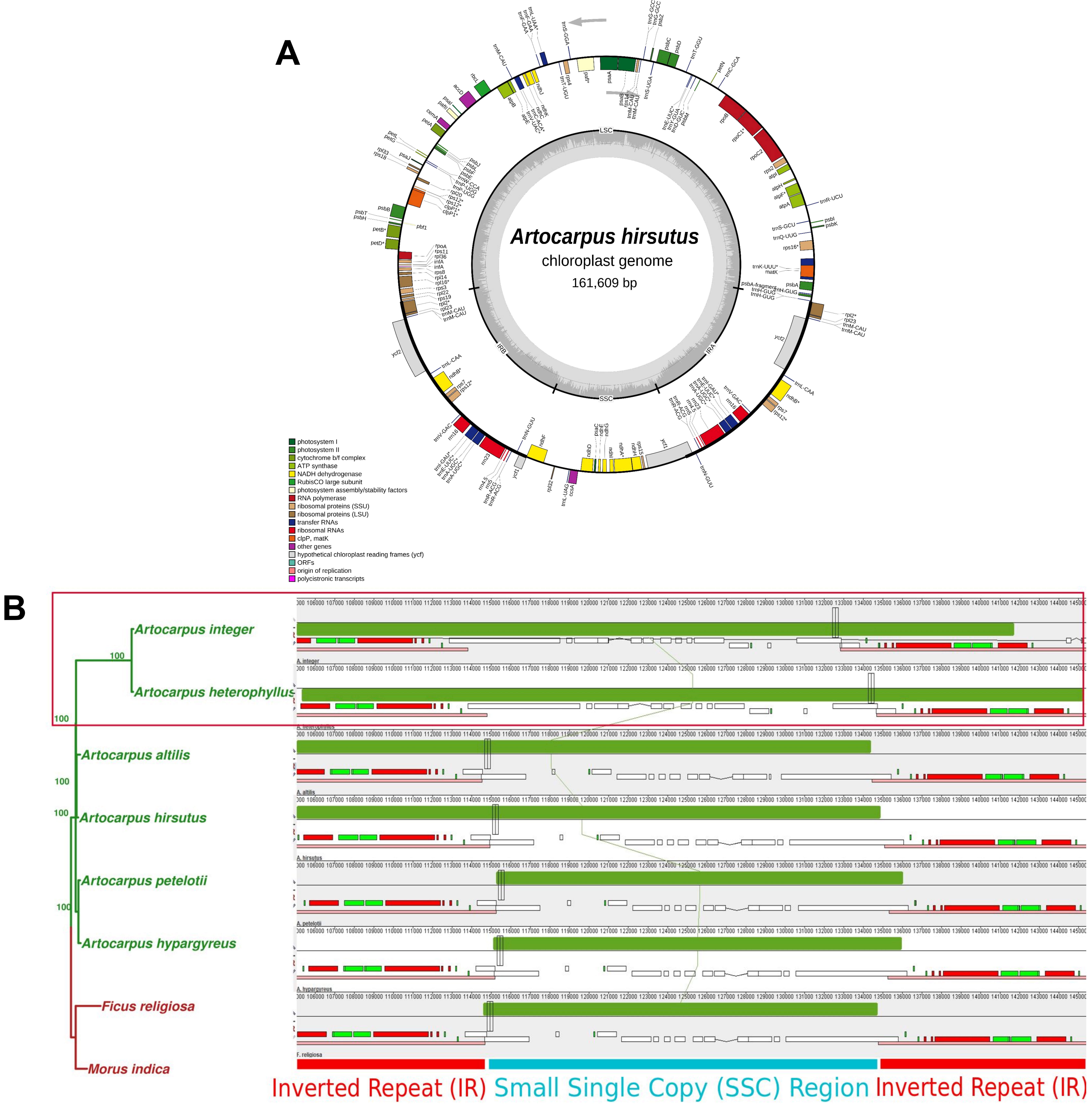

Supplementary Figure S3

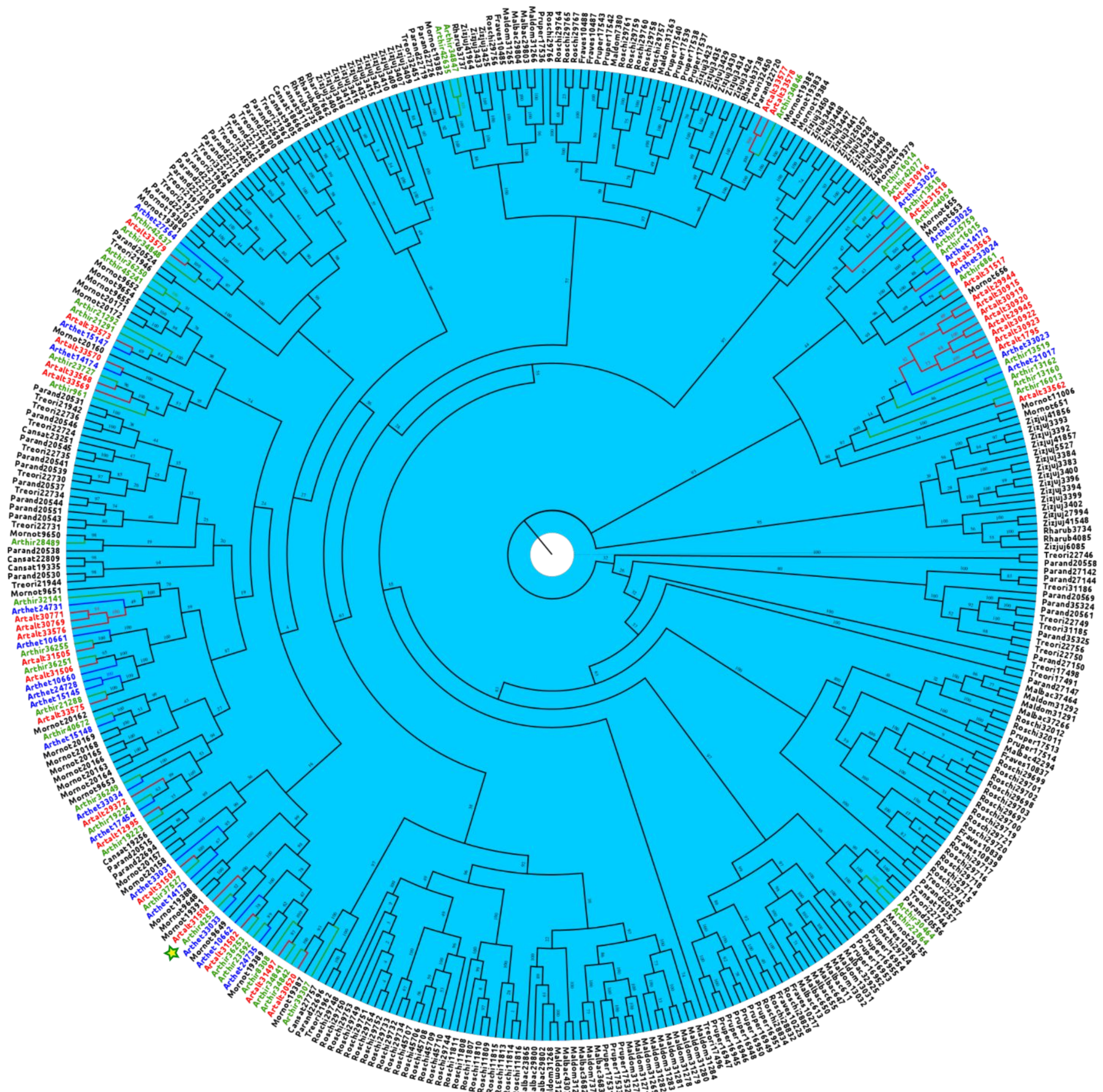

Supplementary Figure S4

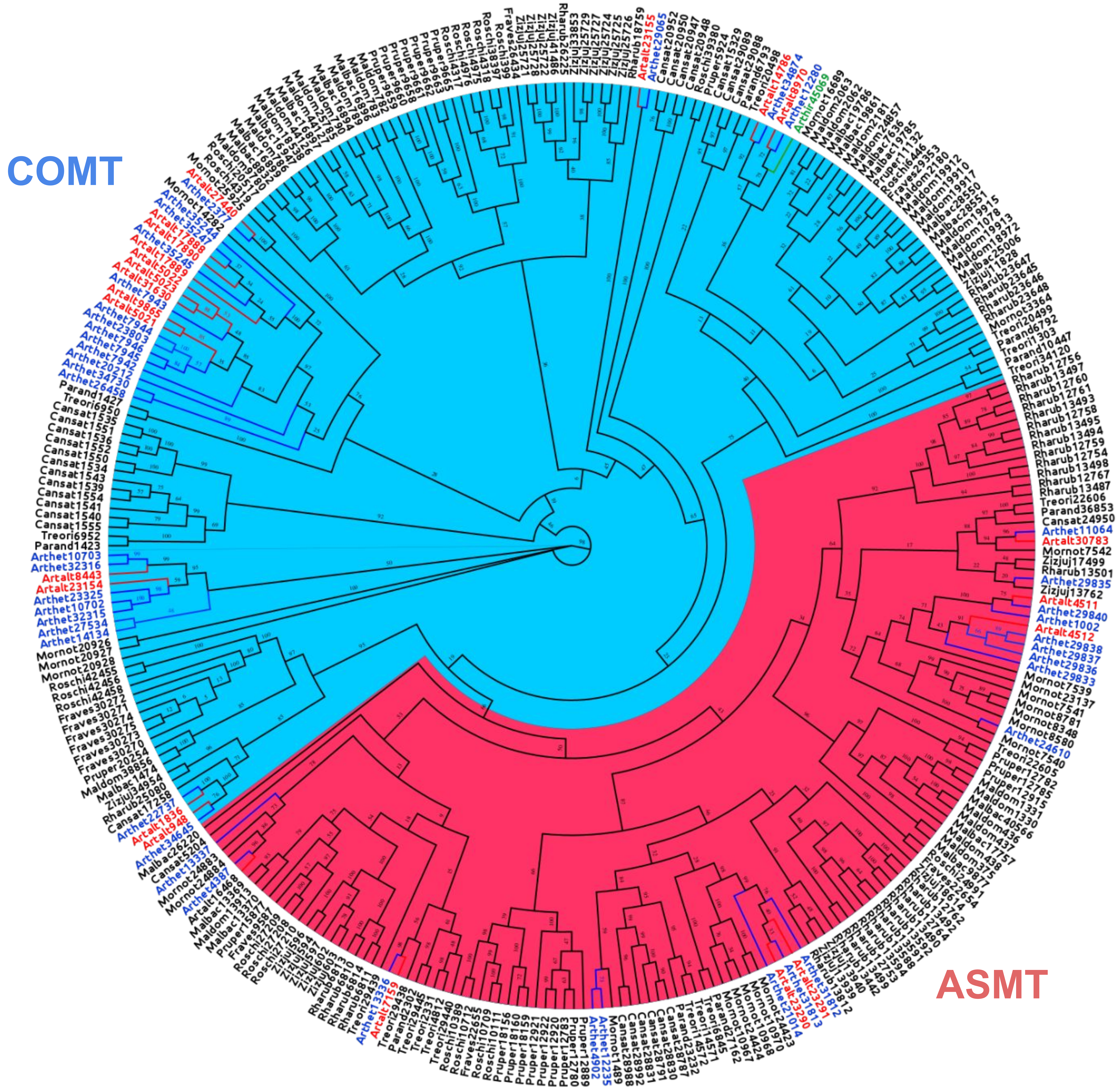

Supplementary Figure S5

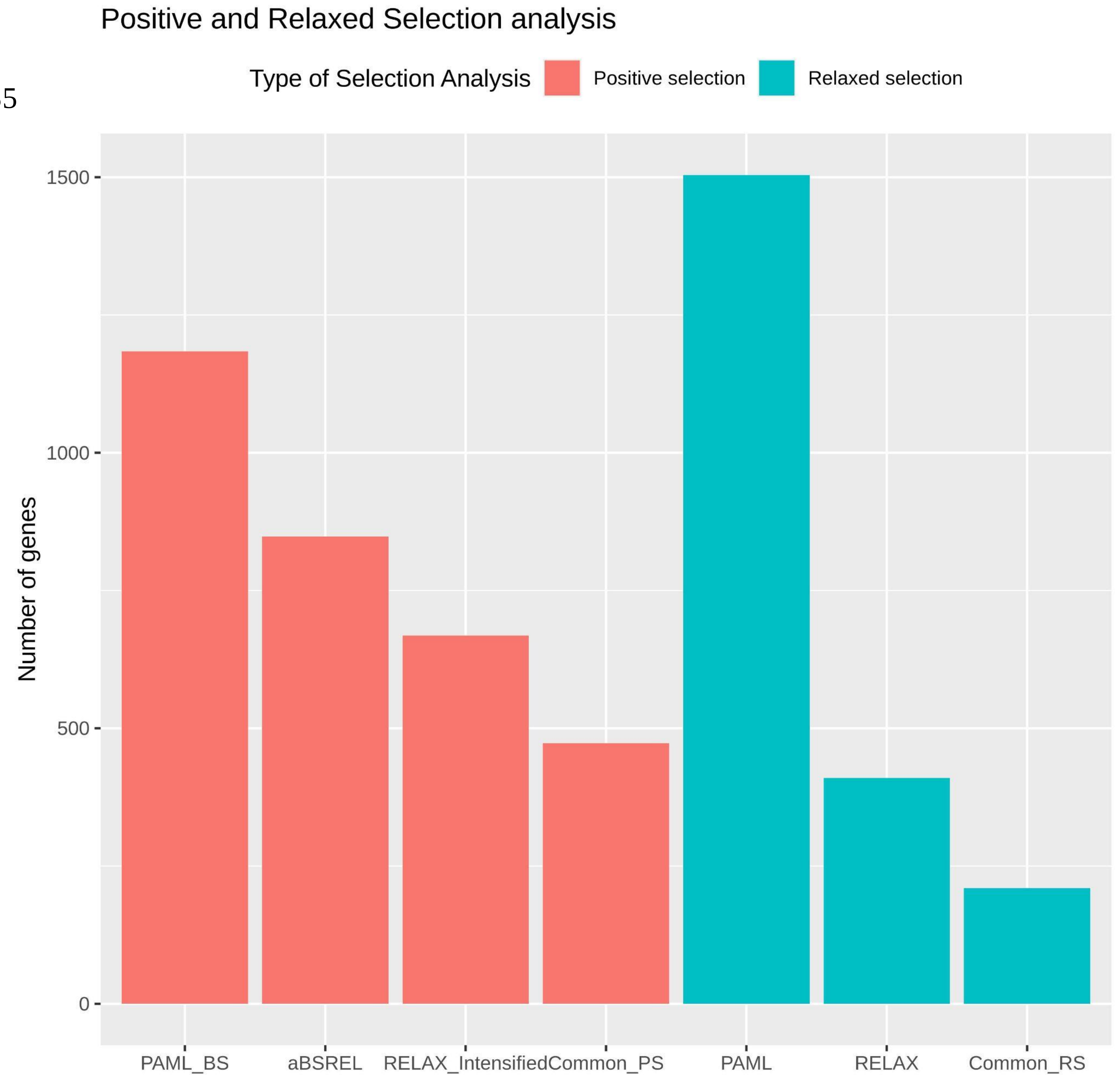

Supplement: Supplementary Figure 1 — Leaf specimen of A. hirsutus (Wild Jack) sampled from the sacred groves, Kodagu, Coorg, Karnataka, India. [file DataSheet_1.pdf]
